# 

**Published:** 1913-07-15

**Authors:** 


### ANNOUNCEMENTS.

First Pan American Dental Congress, Rio de Janeiro, Brazil, October, 1913. Rio de Janeirc, by many considered to be the most beautiful capitol in the world, is, in the coming month of October, to be the seat of the first Pan-American Dental Congress, an event of scientific importance which should mark an epoch in the progress of our profession.

We want your support, but more than that, we want your company. We are inviting you as a guest, and as such the expenses of your stay will be defrayed by the Congress.

Can you not, instead of going to Europe for the seventh time, come to Brazil for the first? You would have an opportunity to gaze upon the most beautiful and fantastic scenery, enjoy an unrivalled climate, and witness the astounding progress of what is, after all, an American city.

Whether you are dentist or manufacturer, our Congress should attract you, the scientific interest of its deliberations is assured by the professional standing of the members of its Commission—its organization is under the official auspices of the Brazilian Minister of Foreign Affairs and others—and it will be the means of bringing together a large number of keen, wide-awake dentists from all the Americas who are anxious to get acquainted with any novelties that might be exhibited.

The work of the Congress will be divided into five sections:

1. Biology, and Related Sciences.
2. General Clinical Work.
3. Prosthesis and Orthodontia.
4. Electricity and Photography as applied to Dentistry.
5. History, Bibliography and Dentology.

These sections will comprise studies of Anthropology, Ethnology, Comparative Anatomy, Pathology and Palaeontology.

The building in which the Congress will be held, the Monroe Palace, is admirably adapted for the purpose—a beautiful edifice affording ample space for sessions and exhibits.

The Congress has for its commission a group of professional dentists who are right on the spot, this does not prevent it from being thoroughly Pan-American, whether you are from California or Cuba, Arkansas or Argentina, come and you will be made welcome.

Further details regarding the Congress, as well as information for transportation facilities, hotel accommodation, etc., may be obtained from Mr. Reginald Gorham, 4727 Hazel Ave., West Philadelphia, Pa.

PROF. R. DE PEREIRA E MAIA,
*President of the Central Commitee.*
RUA GONCALVES DIAS 82.
Rio de Janeiro, Brazil.

THE PANAMA-PACIFIC DENTAL CONGRESS. The work of promoting the Panama-Pacific Dental Congress is progressing in a most satisfactory manner and the Committee of Organization is pleased to report that up to date, in thirty-eight states of the United States, Executive Committees have been appointed to co-operate with it in advancing the interests of the Congress.

Seventeen foreign states and countries and the Island possessions of the United States have taken similar action and the appointment of like committees in other states and other countries are now pending.

It is hoped that within the next three or four months every state and country in the world, where dental organizations are known to exist, will be represented by an Executive Committee, the duties of which will be to promote an interest in the Congress and secure memberships and contributions to the program.

Instructions defining the duties and authority of these committees will shortly be sent to each of them.

The Committee is in almost daily receipt of letters from all parts of the world promising support and attendance and from present indications the Congress will be the greatest yet held by the Dental Profession.

Dr. H. A. Frederick, of San Francisco, has been elected to fill a vacancy in the Board of Directors and the Committee of Organization, in order to promote the interests of the Congress and avoid a repetition of the unpleasant controversies and contests for office which disturbed the organization and opening of the Fourth International Dental Congress, has effected a permanent organization with the following officers:

President, Dr. Frank L. Platt, San Francisco, Cal.
Vice-President, Dr. Charles M. Benbrook, Los Angeles, Cal.
Secretary, Dr. Arthur M. Flood, San Francisco, Cal.
Treasurer, Dr. Fred G. Baird, San Francisco, Cal.

Executive Committe, including the officers named above:

Dr. J. Loran Pease, Oakland, Cal.
Dr. H. G. Chappel, Oakland, Cal.
Dr. R. B. Giffen, Sacramento, Cal.
Dr. A. M. Barker, San Jose, Cal.
Dr. Arthur W. Chance, Portland, Ore.
Dr. G. T. Williams, Seattle, Wash.
Dr. Geo. F. Stiehl, Salt Lake City, Utah.
Dr. B. M. Brookfield, Idaho Falls, Idaho.
Dr. H. H. Wilson, Phoenix, Ariz.
Dr. H. A. Frederick, San Francisco.
Dr. W. A. L. Knowles, San Francisco.

Nearly $13,000.00 has now been subscribed to the promotion fund, the Southern California Dental Association at its recent meeting having subscribed $1,000.00, and its members as individuals, nearly $1,500.00 more.

The Pacific Coast States, aside from California, represented on the Board of Directors, are doing their part and the success of the Congress, so far as can be judged at the present time seems positively assured.

